# From Extraction to Valorization: Unlocking the Potential of Bark-Derived Extraction Residues for Sustainable Material Development

**DOI:** 10.3390/molecules30234537

**Published:** 2025-11-24

**Authors:** Julia Dasiewicz, Anita Wronka, Grzegorz Kowaluk

**Affiliations:** Institute of Wood Science and Furniture, Warsaw University of Life Sciences—SGGW, Nowoursynowska St. 159, 02-776 Warsaw, Poland; julia_dasiewicz@sggw.edu.pl

**Keywords:** bark, cascade processing, extraction residues, bark extraction, biorefining, upcycling potential, post-extraction, waste, biomass

## Abstract

Tree bark, a renewable byproduct of the forest industry, has long been recognized as a rich source of bioactive and structural compounds, including polyphenols, tannins, triterpenes, and suberinic acids. Over recent decades, numerous studies have explored bark extraction processes aimed at recovering these valuable substances. However, the substantial quantities of solid and liquid residues remaining after extraction are still largely overlooked despite their significant potential for further valorization. This review summarizes the current state of research on bark extraction, highlighting the diversity of applied techniques from conventional solvent extraction to advanced green methods such as organosolv, subcritical water, and supercritical CO_2_ extraction. Particular emphasis is placed on post-extraction residues, which remain rich in lignocellulosic, suberinic and phenolic compounds suitable for the development of bio-based materials, composites and functional chemicals. Importantly, this review introduces a novel perspective by evaluating post-extraction residues with the same significance as primary bark extracts, emphasizing their un-tapped potential within emerging bark biorefinery concepts. The review identifies existing knowledge gaps related to the chemical characterization, recovery strategies and industrial integration of these byproducts. Finally, it outlines future research directions focused on transforming bark extraction residues into high value sustainable materials fully aligned with the principles of the circular bioeconomy and zero waste processing.

## 1. Introduction

**Figure 1:**
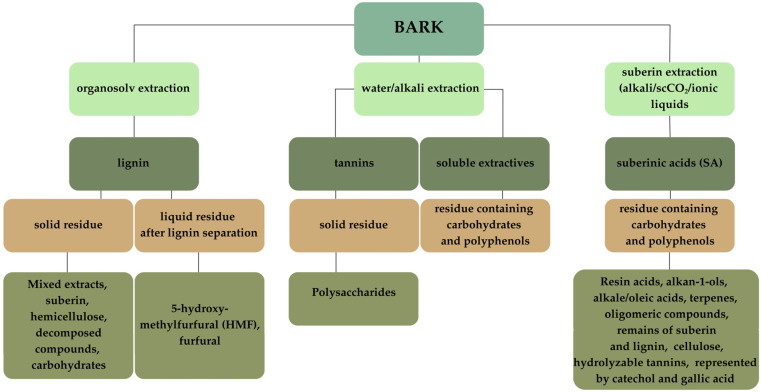
Illustration of the current state of knowledge about bark post-extraction waste (own elaboration).

The circular bioeconomy represents a fundamental shift from linear models of production and consumption toward regenerative, closed-loop systems that emphasise the sustainable use, recycling, and recovery of biological resources. Globally, this concept is gaining momentum as a strategic response to climate change, resource depletion, and the pursuit of sustainable economic growth. Within this framework, the forest-based sector plays a pivotal role, as wood biomass constitutes a renewable raw material with significant potential for cascading use extending product lifecycles, promoting material reuse, and enabling energy recovery at the end of use. Despite this potential, current industrial practices often prioritise bioenergy generation over the high-value valorisation of wood byproducts, such as tree bark. Yet, bark remains a chemically rich and largely untapped source of bioactive compounds and lignocellulosic materials, offering considerable opportunities for innovation in materials science, biotechnology, and green chemistry [1,2,3]. The chemical complexity of bark enables diverse valorisation pathways, often initiated by specific extraction techniques designed to isolate high-value components, such as lignin, tannins, or suberin (see Figure 1 for an overview of potential extraction pathways and products). Although bark accounts for an average of around 10%, or even 10–20%, of the trunk’s volume, it is often treated as waste material in the timber industry [4,5]. Its annual production is enormous, reaching approximately 17 million m^3^ in Canada and over 10 million m^3^ in Sweden and Finland, with the main source of this waste being the debarking process [5,6,7]. Forests and other wooded areas cover over 43.5% [8] of the EU’s land area, and their main function in the European Union is the production of wood, which is usually debarked [9]. The recently completed REHAP project (EU H2020-SPIRE) estimated that 23 million tons of wood bark are available as lignocellulosic material after debarking of both coniferous and hardwood species [10]. These data come from 2018; however, the same project predicts that by 2027, the amount of bark obtained only from spruce and pine species will exceed 15 million tons [11], not to mention all other wood species. At the policy level, the European Green Deal and the EU Bioeconomy Strategy have positioned the European Union as a global leader in the transition toward a circular bioeconomy [12,13,14,15]. These frameworks aim to achieve carbon neutrality by 2050 by integrating climate, biodiversity, and resource-efficiency objectives within industrial and energy policies [14]. They promote the sustainable use of biomass across energy, construction, and transport sectors, while encouraging the valorisation of forestry byproducts, including tree bark, to reduce waste generation and enhance material circularity [12,13,14,15]. The EU Bioeconomy Strategy further emphasises innovation in bio-based industries, the sustainable management of natural resources, and the resilience of production systems to climate and supply risks, highlighting the need to optimise the use of non-food biomass such as bark for bioenergy, biochemicals, and advanced materials [12,13,15]. In support of these goals, the EU has introduced regulatory and financial instruments that foster decentralised biowaste management, stimulate investment in bio-based value chains, and strengthen the competitiveness of the European bioeconomy [14]. Nevertheless, gaps remain in aligning carbon-sink targets with the increasing intensity of biomass extraction, underscoring the need for balanced policies that ensure both environmental integrity and economic viability [12,13,14,15]. EU policies increasingly emphasise the importance of balancing bark biomass extraction with the preservation of biodiversity and ecosystem services. To achieve this, the European Union promotes sustainable forest bioenergy pathways that mitigate carbon emissions while maintaining healthy ecosystem conditions, for example, by limiting the removal of fine woody debris and protecting soil functions [16]. These initiatives are closely linked to the principles of the circular economy, which call for stricter resource extraction practices and regenerative approaches that restore ecological balance and enhance forest resilience [17]. Nevertheless, policy gaps remain in effectively integrating biodiversity considerations into land-use planning and biomass extraction strategies, particularly in regions with intensive forest utilisation [18]. Addressing these gaps is crucial to ensure that bark valorisation and biomass-based industries contribute not only to climate and energy goals but also to the long-term stability of forest ecosystems. Challenges in data collection and monitoring constrain the effective utilization of bark biomass. National forest damage survey programs are often fragmented and inconsistent, hindered by legal frameworks and language barriers, which complicates cross-border assessment and comparison. Additionally, there are notable regional disparities in data openness and harmonization, limiting the ability to make informed policy and management decisions [19]. While emerging biorefinery technologies and wood fractionation methodologies show promise for enhancing the valorisation of bark, their current implementation remains limited in scope [20]. Taken together, these issues underscore that although EU policy evolution reflects a growing commitment to sustainable bark biomass utilisation, persistent gaps in regulatory alignment, data harmonisation, and the integration of biodiversity considerations must be addressed to realise the objectives of a circular bioeconomy fully. Currently, more than half of the collected bark is burned or stored in landfills, while the remainder is mainly used as a cheap source of energy in sawmills and pulp mills. Both burning, which can lead to pollution and damage to combustion chambers due to the high ash content and lower sintering temperature compared to wood ash, and bark storage are associated with environmental problems [7]. However, the growing demand for lignocellulose sources and the shortage of forest land are prompting intensive research into the optimal use of bark [4]. The bark contains a large fraction of extractives and lignin, accounting for up to 50% of the dry weight, which represents a valuable renewable source of chemicals, particularly aromatic compounds [7]. Numerous scientific studies confirm its potential, highlighting its growing importance in various industries worldwide [4].

Bark waste in Poland presents both a challenge and an opportunity for environmental management and resource utilisation. Its potential for use is significant, especially when we consider wood waste from consumers and its impact on pollution monitoring. In 2013, approximately 6.5 million cubic meters of wood waste, including bark, were generated in Poland. The main source of this waste was the construction sector, accounting for 64% of the total, primarily due to the use of wood products such as windows and doors [21]. Recent studies from the last five years (2020–2025) highlight the increasing interest in measuring and valorizing bark and bark by-products across Europe. Although it is an important forestry by-product, bark has not been captured in official waste statistics owing to a lack of shared and harmonized reporting. Global bark generation is estimated at 300–400 million m^3^ per year, with a considerable share in Europe [22,23]. In Poland, although it is not possible to deliver bark-specific data, general waste recovery rates remain around 48%, with 42% of total waste still sent to landfills [24,25].

Across Europe, reuse and valorization rates vary substantially: frontrunner countries such as Germany, Sweden, and Italy report recycling rates exceeding 35% and landfill shares below 1%, while others, including Cyprus and Malta, still landfill over 40% of their organic residues [26,27,28].

Despite these trends, a clear gap exists in terms of comprehensive life cycle inventories and techno-economic assessments, which are specific to feedstock. The LCA/TEA studies that have been integrated across each of the systems to date have focused on the emissions reduction potential associated with bark valorisation—specifically when low-carbon emission energy sources and solvent recycling are used in the processing [29,30,31]. Still, methodological gaps are still common practices, and there is inequalities among emissions and lack of transparent data regionally, thus hindering informed decision-making and policy development in bark residue management [23,32,33].

Furthermore, tree bark, such as that of *Betula pendula*, has been used in biomonitoring studies to assess heavy metal pollution in various forest areas in Poland. Seasonal fluctuations in heavy metal concentrations in bark indicate its effectiveness as a bioindicator, with higher levels observed in winter [34]. Research also shows that bark and wood waste can be effectively utilised through mycological degradation, using specific fungi to convert waste into valuable products [35].

Post-production waste from bark, particularly that of birch and willow, presents significant opportunities for sustainable applications across various industries, actively contributing to the development of a circular economy. Residues from extraction processes, such as suberinic acid or willow bark waste, can be effectively reused. Birch bark residues from the extraction process represent a valuable raw material with a wide range of applications, especially in the production of particleboard, where they can serve as both a filler and a binder. Adding up to 10% of these residues significantly improves the physical and mechanical properties of the boards while maintaining compliance with European standards. The composition of birch bark residues, including suberic monomers, lignin, cellulose, and esters, makes them ideal for use as fillers in particleboard [36]. Moreover, these residues can be utilised as an innovative, formaldehyde-free binder in wood-based composites, particularly particleboard, which significantly enhances the ecological profile of the product and reduces dependence on synthetic adhesives. Research confirms that their addition, up to a certain threshold, also improves the mechanical properties of the material [37,38]. In addition to its applications in the wood industry, birch bark is also a rich source of bioactive compounds with antioxidant and antibacterial properties, which can be isolated and used in pharmaceuticals and food preservation [39]. Alternatively, bark residues can be processed to produce energy, carbon sorbents, and other bioproducts, highlighting their versatile valorisation potential beyond traditional applications. Optimizing methods, including the combustion of birch bark residues, significantly increases the efficiency of this process [40]. The environmental benefits of this approach are significant: using bark waste minimizes its volume and promotes sustainable industrial practices [41]. In turn, willow bark residues, rich in organic matter, are ideal for composting, significantly improving the nutritional profile of sewage sludge from municipal treatment plants and supporting better decomposition of organic matter and nutrient cycling [42]. Despite its promising potential, challenges remain in optimising processes to maximise efficiency and the quality of end products. Further research is needed to utilise these valuable materials fully.

The purpose of this publication is to provide a comprehensive review of current research on the utilization and valorization of bark across different sectors of the economy. This review aims not only to identify well-established and underexplored research areas but also to highlight technological challenges and opportunities relevant to the large-scale implementation of bark-based solutions. In the context of the European Green Deal and the transition towards a circular bioeconomy, such valorization pathways are essential for enhancing carbon neutrality and resource efficiency.

The literature review presented in this article also provides the foundation for further research conducted under the BarkPRO project, funded by the Polish National Science Centre (NCN) within the OPUS programme. This project focuses on basic research and aims to determine the properties and processing potential of tree bark, particularly in the context of post-extraction residues. At this stage, the research is not directed toward practical implementation or commercialization but is intended to deepen our theoretical understanding and identify fundamental relationships necessary for the future development of bark-based biotechnological applications.

## 2. Chemical Composition of Tree Bark: Extraction Potential

Tree bark represents a valuable lignocellulosic resource, composed mainly of polyphenols (approximately 45% of dry weight), polysaccharides (around 50%), and a smaller fraction of inorganic compounds and lipophilic extracts (about 5%) [43]. 

Different species exhibit distinct chemical profiles; for instance, the bark of *Pinus radiata* shows variations in phenolic and carbohydrate components depending on tree height [44], while white birch species (*Betula resinifera, B. pendula, and B. platyphylla*) display pronounced species-specific chemical differences that become more distinct with age [45]. A strong phylogenetic signal in bark density and composition further highlights the role of evolutionary history in shaping these traits [46]. Ontogenetic changes also contribute to variability, as young birch saplings exhibit greater chemical diversity than seedlings, with profiles that differentiate as the trees mature [45]. In *Abies alba*, the concentration of polyphenolic compounds varies longitudinally within the stem, with higher concentrations observed below the crown [47]. Environmental conditions also exert a significant influence: geographic location and climate drive chemical variation, such as the seasonal and habitat-dependent production of phenolics in *Libidibia ferrea* across the Caatinga and Atlantic Forest biomes [48]. Trees also modify their bark chemistry in response to stressors like drought, temperature extremes, and soil salinity, often increasing secondary metabolites such as flavonoids and tannins to enhance stress resistance [49,50]. In urban environments, pollution can alter bark pH, thereby influencing epiphytic lichen colonization, although bark pH appears to be less important than species identity [51]. Functionally, bark fulfils protective roles, including defence against fire and water stress; for example, tree species in dry tropical forests develop thicker bark with specialised anatomical features to withstand harsh conditions [50]. Finally, bark decomposability and flammability vary widely across species, largely driven by chemical traits such as lignin concentration, which is a key determinant of thermal stability, resistance to microbial degradation, and fire-retardant properties—factors crucial for both ecological resilience and material applications [52]. A comparison of the structure and chemical composition of different species in terms of their extraction potential is presented in Table 1.

The extraction of bioactive molecules from bark has been the subject of research in recent years, leading to the development of multifunctional products that, although obtained with low efficiency, are characterized by high added value [80]. The low extraction yield results in significant amounts of post-extraction residues that remain rich in valuable compounds. Numerous studies have been conducted on the isolation and characterization of bark components, including tannins [10,43,81,82,83], lignin [84,85,86], cellulose [87,88,89], and suberin [90,91,92,93,94,95,96,97,98]. The relationships between these major groups of bark constituents, their key functional properties, and high-value application areas are summarized in Table 2.

Tree bark structure varies widely among species; for instance, differences in bark thickness, density, fissuring, and the proportion and arrangement of tissue types such as phloem, sclereids, and fibers reflect adaptations to environmental pressures. These anatomical variations correlate with differences in chemical composition, such as levels of lignin, extractives (e.g., polyphenols, triterpenes, and sterols), and suberin, which in turn affect the extraction potential of bioactive compounds [121,122]. Dense, lignified bark with high suberin content typically requires more aggressive or multi-stage extraction methods, whereas species with porous, thin bark enable more efficient solvent penetration and compound recovery [123]. Overall, bark from different species demands tailored extraction protocols that account for these structural and chemical differences, as the interplay between anatomy and chemistry largely determines extractive yield, selectivity, and compound stability. These anatomical–chemical relations build upon the innate chemical composition of bark, which is governed by the amounts of lignin, cellulose, hemicelluloses, suberin, tannins, and other phenolic resources, that better dictate the selection and efficiency of extraction techniques. Species that contain substantial amounts of hydrophilic polysaccharides in their bark may be easier to extract in an aqueous or enzymatic system, whereas species that are predominantly composed of lipophilic extractives, suberin, or complex phenolic fractions may then require organic solvents, deep eutectic solvents, or supercritical CO_2_ extraction to recover these compounds efficiently. Ultimately, differences in composition dictate extraction selectivity and yield, and compounds that affect the chemical profile of the solid residues after extraction will also modify the potential for reuse pathways through material applications, as well as adsorbents and/or precursors for bio-based chemicals. Thus, the chemistry of bark, the extraction method, and the properties of the residual material provide a basis for the next explanation of the extraction technologies that follows.

## 3. Utilization Strategies for Tree Bark as a Byproduct Value

### 3.1. Materials Industry

The use of bark in the production of wood-based boards offers a way to a more sustainable industry. This method allows for waste management and reduces the consumption of precious raw materials. A comparison of bark utilization strategies in different sectors is presented in Table 3. Bark, a by-product, is rich in phenolic compounds, including tannins and polyphenols, which can serve as natural binders, replacing synthetic adhesives such as phenol-formaldehyde and urea-formaldehyde [90,91]. The use of bark in the production of particleboard promotes resource optimization and sustainable development in the wood industry [92,93].

However, integrating bark into boards has both advantages and disadvantages. Studies show that although bark can increase water absorption and swelling thickness [147], white birch bark has hydrophobic properties, which make it suitable for improving the water resistance of the outer layers of boards [148]. It is also possible to optimize the density of the boards to the desired level, e.g., approximately 750 kg m^−3^ [127]. On the other hand, an increase in bark content often leads to a decrease in mechanical strength, which manifests itself as a significant reduction in the modulus of rupture (MOR) and modulus of elasticity (MOE) compared to boards made completely of wood [147]. The strength of the internal bond is also negatively affected by an increase in the content and size of bark particles [149].

The primary challenge in utilizing bark is to strike the right balance between maximizing the quantity of this raw material additive and preserving the necessary mechanical properties of the boards. In-depth research indicates that the reduction in strength is notable: incorporating 25% Douglas fir bark led to a drop in the MOE, MOR, and internal bond strength (IB) by 20% to 30%, while also causing an increase in linear expansion (LE) of almost 25%. This reduction is mainly due to the lower cellulose content in bark compared to wood, which diminishes the bonding strength. To mitigate this adverse effect, it is essential to refine the geometry of the bark particles and their pre-treatment process. Research on black spruce bark has demonstrated that enlarging the size of the bark particles (e.g., to 5.0–7.0 mm) considerably reduces the internal bond strength (IB), whereas other investigations on extracted black spruce bark indicate that smaller particles may necessitate further optimization to reach the desired IB strength, which remained low (e.g., 0.49 MPa) even at the ideal size. The ability to satisfy standards is closely tied to the bark content: boards containing 30% spruce bark were deemed suitable for furniture manufacturing, while those with 50% black spruce bark content met the ANSI A208 standard. Nevertheless, their MOE and MOR were reduced by 12% and 37%, respectively, compared to the control boards. None of the panels made entirely of bark met the minimum strength criteria (MOR), highlighting that bark functions better as a partial substitute rather than a complete raw material for structural applications [127,147,148].

Furthermore, insulation boards made from bark provide enhanced thermal conductivity and heat storage capabilities, making them appropriate for applications in civil engineering [128]. Bark panels show remarkable sound absorption qualities, exceeding those of standard materials. The incorporation of tannin-based adhesives further enhances their acoustic performance, making them ideal for noise reduction. This endeavor is closely associated with the advancement of innovative adhesives free from formaldehyde. Utilizing tannins and polyphenols derived from bark is essential for creating environmentally friendly, formaldehyde-free adhesives that prevent the release of harmful Volatile Organic Compounds (VOCs) [129]. In the realm of synthetic adhesives like phenol-formaldehyde (PF) resins, pre-extracting bark with hot water is vital as it removes low molecular weight substances that hinder the curing of PF resin, thereby enhancing the quality of bonding. Recent developments in this area involve utilizing bark plasticization and high-temperature polymerization of its extracts to promote particle self-bonding, which is critical for manufacturing materials without the use of traditional binders [5,130]. Contrary to the prevailing negative trends, research on *Acrocarpus fraxinifolius* boards has indicated that incorporating 30% bark did not negatively impact MOR and MOE and, in some instances, enhanced physical properties, leading to reduced swelling after 24 h [148]. Additionally, bark can be transformed into porous materials through mechanical foaming, enabling the creation of structures with varying properties. These materials find applications in insulation and beyond, showcasing the adaptability of bark as a raw material [130].

### 3.2. Farming and Gardening

The use of tree bark waste in farming and gardening is a sustainable approach to managing forestry by-products. Bark, often treated as waste, can be transformed into precious substrates and soil improvers, supporting plant growth and improving soil structure. This method not only addresses waste management issues but also promotes sustainable agricultural practices. *Eucalyptus globulus* and *Pinus radiata* bark, when properly water-treated, can be used to improve seed germination. A mixture of 75% extracted E. globulus bark fiber with commercial substrates such as peat and coconut fiber showed promising results in stimulating the growth of radishes and Chinese cabbage. The addition of phytostimulants, such as fulvic acid in capsules, further promoted plant growth, demonstrating the potential of bark-based substrates in gardening [131]. Tree bark can be used as a gardening substrate, offering many benefits such as nutrient richness [35] and improved aeration and drainage, which is crucial for root health [132]. However, certain challenges should be kept in mind: high initial concentrations of soluble salts in the bark can be toxic if not properly rinsed [132], bark waste can carry pathogens or contaminants that negatively affect plant health [150], and its physical and chemical properties can vary significantly, leading to inconsistent performance [151]. Crushed bark, particularly spruce bark, has been tested as a soil improver in organic farming. The application of crushed bark with base ash for one year significantly increased wheat yields and quality, demonstrating its effectiveness in enhancing soil fertility and crop productivity. The high C/N ratio in shredded bark suggests its potential to enhance soil organic carbon content and cation exchange capacity. However, further research is necessary to fully understand its long-term effects [133].

### 3.3. Energy Sector

It is worth emphasising that, generally speaking, bark is classified as an industrial residue, most often burned for energy production or discarded in rural areas without being given any value-added use [87]. However, the use of bark waste as biomass is a promising method of energy production that contributes to reducing greenhouse gas emissions [134]. Several key technologies enable the effective processing of bark.

Chemical looping combustion (CLC) technology offers an efficient and cost-effective approach to utilizing bark from the pulp and paper sector. Research indicates that a CLC setup employing ilmenite as an oxygen carrier can exhibit low thermal expenses and has an estimated payback period of around 9.69 years. This technology also shows a potential for reducing CO_2_ emissions by about 90% compared to conventional combustion methods, which is a significant benefit for sustainability [134]. Additionally, bark can be subjected to gasification in two-fluid steam gasifiers, enabling the generation of synthesis gas and, consequently, thermal and electrical energy. Dual Fluidized Bed (DFB) gasifiers, utilizing steam as the gasifying medium, offer notable benefits by generating nitrogen-free syngas with a high calorific value, typically ranging from 12 to 14 MJ m^−3^. This nitrogen-free syngas is exceptionally advantageous not only for generating heat and power but also for chemical synthesis and biofuel production [136]. Ahlström et al. conducted research showing that bark used in double fluidized bed gasifiers can yield gas quality on par with that of wood pellets. Investigations confirmed that after reaching optimal moisture content (~10%), the gasification of bark in DFB achieves a biomass-to-gas conversion efficiency of 75%. Following this, the efficiency of converting this gas into biomethane can achieve 65%. Bark in DFB gasifiers attained a cold gas efficiency of 70%, comparable to the efficiency achieved with wood pellets. Utilizing bark instead of pellets as a lower-value feedstock leads to a reduction in raw material costs by 30%–40%, significantly enhancing the economic feasibility of energy production [137]. Moreover, bark can be directly burned in steam boilers, showcasing a high calorific value and impressive energy efficiency. Studies focused on bark combustion in a steam boiler indicated an energy efficiency of 98.24 × 10^6^ BTU/h (British Thermal Units). A critical aspect of process efficiency involves keeping the fuel's ash content low (under 1%) and ensuring the bark is adequately dried before combustion. The efficiency of burning bark is similar to that of burning wood chips [135], making it an appealing alternative in existing energy systems. Additionally, bark waste can be combined with plastics and oil to create energy-dense briquettes. Research demonstrates that blending bark with plastic and oil waste can yield briquettes with a calorific value of up to 33.56 MJ kg^−1^ for the optimal mixture (30:70:100, plastic:oil:bark). This significantly surpasses the average energy content of coal (15.0–27.0 MJ kg^−1^) and dry wood, meeting the European standard EN 14961–3 (ENplus A2). This method not only aids in waste management but also serves as an alternative fuel source, thereby lessening reliance on fossil fuels [138]. The inclusion of bark in mango wood improves its energy characteristics, making it a compelling fuel option; however, in other species, an excess of bark can diminish efficiency [152].

### 3.4. Dietary Supplements, Cosmetics, and Pharmaceuticals

Tree bark, a byproduct of the forestry sector, is prized for its high content of polyphenols and flavonoids, making it suitable for creating dietary supplements and cosmetics [141]. Extracts from tree bark possess potent antioxidant capabilities (for instance, those derived from spruce and pine) [124,125] and can effectively block UV radiation [140]. Research indicates that extracts from Scots pine bark typically contain a greater concentration of polyphenols (such as quercetin, eriodictyol, and (–)–epicatechin), whereas English oak extract is abundant in cardioprotective substances like naringenin [125]. In contrast, larch (*Larix*) bark has a significant presence of high molecular weight proanthocyanidins, which can be transformed into effective UV blockers for cosmetic uses through innovative techniques like catalytic hydrogenolysis in ionic liquids [140]. Utilizing bark aligns with the principles of a circular economy.

While polyphenols hold potential for various applications, their commercialization encounters three major challenges: (1) Securing regulatory approval requires more extensive evidence on both the short-term and long-term effects of polyphenols, as well as a deeper understanding of their diversity and bioavailability [141]. (2) There is a necessity for standardization in extraction processes, which must address the variability in chemical composition influenced by species and harvesting conditions by developing efficient, cost-effective, and reproducible extraction methods, including innovative techniques like hydrogenolysis [140,141]. (3) The development of products necessitates the refinement of the final formulation (for instance, by combining different extracts) and the use of proprietary manufacturing processes to achieve high levels of active compounds. Moreover, alternatives should be explored for the solid waste produced after extraction, such as transforming it into fuel pellets [141].

### 3.5. Lignin as a High-Value Component of Bark Residues

Bark-derived lignin represents a structurally distinct and increasingly valuable component of bark residues, and its high-value utilization has become a major research focus. Recent studies highlight several emerging extraction strategies, including deep eutectic solvents enabling high-purity lignin recovery from lignocellulosic biomass [142], microwave-assisted extraction optimized for phenolics and tannins as part of greener processing routes [153], and solvolytic liquefaction yielding lignin-derived bio-polyols for advanced materials [143]. Compared with wood lignin, bark lignin is typically richer in phenolic structures [144], more heterogeneous [145], and contains abundant hydroxyl functionalities that facilitate chemical modification, for example, in the synthesis of non-isocyanate polyurethanes (NIPUs) with tunable performance [145]. These structural features open pathways to a wide range of high-value applications, including lignin-derived polymers such as rigid foams [142], natural adhesives replacing petroleum-based systems [146], antioxidants due to its high phenolic content [144], and bio-based chemicals produced through catalytic depolymerization [154]. Overall, recent advances demonstrate the growing potential of bark lignin as a versatile feedstock for sustainable materials and chemicals, supporting its strategic role in bark-based biorefinery concepts.

### 3.6. Solvent Recovery and Recycling Considerations

Solvent recovery is a vital consideration both for environmental and economic sustainability when comparing bark-extraction processes, particularly for green extraction systems where solvent reuse has a direct impact on environmental sustainability. In terms of processing costs, efficient recycling of CO_2_ in supercritical CO_2_ extraction led to energy savings and efficiencies in reducing operational costs. Current improvements in the processing of supercritical CO_2_ extraction systems are focused on enhancing compressors and recirculation loops to enhance efficiency by minimising energy losses or operational costs [155,156]. In general, solvent recovery will be more difficult for natural deep eutectic solvent (NADES) systems because of the higher viscosity in recovery and the removal and selective destalling of water and solutes from the solvent system; hybrid extraction approaches for the extraction–purification process model have demonstrated a relative improvement in recovery efficiencies, and provided information on solute degradation of solvents [107,157]. Sustainability is improved with sequential and flow-through bioreactor extraction systems due to the adoption of a closed-loop cycle of solvents with little to no losses of the solvent during extraction and purifying lipophilic and polar fractions [158,159]. Integrated biorefinery models are also combining solvent reuse strategies, catalytic, and biological downstream conversion while also reducing waste generation, furthering a circular design process [86,107]. The increase in recent technologies to recover solvents despite continued solvents stability, energy demand, and scale-up, especially with NADES and multi-component solvent systems, requires continued exploration or continuous improvements in a more sustainable, recyclable solvent strategy for industrial bioprocessing applications [105,159].

## 4. Tree Bark Extraction Methods

**Figure 2:**
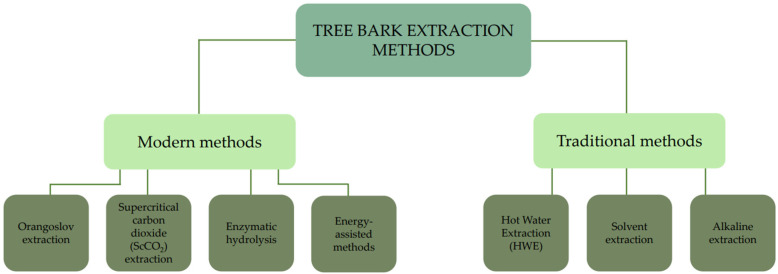
Classification of Tree Bark Extraction Methods.

Bark extraction methods differ in terms of efficiency and environmental impact. One conventional technique is solvent extraction, which utilises organic solvents to isolate desired compounds, such as lignin and tannins. Although this method ensures high extract purity, it raises environmental concerns due to the use of solvents [116,117,160]. An alternative to traditional methods is steam explosion, which involves exposing the bark to high-pressure steam, leading to the release of valuable compounds. This technique is valued for its efficiency and environmental friendliness. Another sustainable method is enzymatic hydrolysis, which uses enzymes to break down the bark, making it suitable for the production of biopolymers. Although it has lower efficiency, its main advantage is that it has no negative impacts on the environment [161]. More modern techniques include microwave-assisted and ultrasonic extractions, which utilise energy to enhance the process efficiency. Studies have shown that microwave extraction yields extracts with higher antioxidant capacity and is an energy-efficient method [162]. The classification of bark extraction methods, divided into traditional and modern, is shown in Figure 2.

The material characteristics of bark in its various derived forms (characterized by varying amounts of lignocellulosic polymers, lipophilic extractives, tannins, and high amounts of suberin) affect the extraction technology selected and the characteristics of the end products obtained from the extraction process. The extraction strategies to extract the polar or non-polar components and compounds will remove a certain class of chemical families, leaving behind higher residues of structural polysaccharides or lignin, or phenolic compounds. The change in composition helps guide the efficiency, effectiveness, and benefits of each extraction method, as well as the pathways of value for solid residues, whether goods (feedstock), adsorbents (bio), biochemicals, or energy. The links in this section provide a basis for a more robust and thorough discussion of multiple extraction technologies. A comprehensive comparison of the standard chemical composition of residues produced by the primary extraction procedures is displayed in Table 4.

### 4.1. Traditional Methods

A detailed comparison of all bark extraction methods, including their extraction medium, environmental impact, and main products/applications, is provided in Table 5.

#### 4.1.1. Hot Water Extraction (HWE)

Hot water extraction is considered an environmentally friendly method. It utilises an accelerated solvent extractor (ASE) with water at temperatures ranging from 100 °C to 160 °C. This method is particularly effective in isolating non-cellulose polysaccharides (NCPs), including hemicelluloses and pectins. The optimal extraction temperature for these compounds, which are particularly rich in arabinose and galacturonic acid, is 140 °C. At this temperature, high-molecular-weight polysaccharides are obtained, and aromatic substances can be partially removed using DAX-8 polyacrylic resin [119,120]. The HWE process offers several benefits that extend beyond the mere extraction of compounds. This method significantly enhances the availability of lignin, which facilitates subsequent delignification processes and enables the recovery of a purer form of this valuable polymer [110]. Additionally, HWE has a positive impact on the quality of biomass, reducing ash content and increasing its calorific value, thereby making the raw material more suitable for energy purposes [111]. In the context of sustainable development, this method enables the efficient production of fermentable sugars and other byproducts, while enhancing the overall quality of biomass [122,123]. Extraction efficiency also depends on the type of bark. Different tree species, such as willow, sugar maple, and Norway spruce, exhibit distinct chemical compositions and properties that influence extraction results [111,112,182]. After HWE, post-extraction waste is generated, comprising residual biomass and by-products. In the case of HWE, the remaining biomass may contain up to 50% ash, as well as unextracted compounds, e.g., phenols [122,123]. By-products such as hemicellulose, pectin, and tannins are also produced, which, if not utilized, pose a challenge for waste management [119,125].

#### 4.1.2. Solvent Extraction

Solvent extraction utilises organic solvents, such as acetone, petroleum ether, or methanol, to isolate lignin and tannins. This method often produces products of higher purity and yield compared to HWE. For example, petroleum ether, followed by acetone, is used to extract tannins from the bark of the Chinese umbrella tree [101]. A key aspect of solvent extraction is the polarity of the solvent, which affects the yield and type of compounds extracted. Polar solvents, such as methanol and ethanol, are effective in extracting phenolic compounds, which possess strong antioxidant properties [163]. Nonpolar solvents, such as isopropyl alcohol, are used for the extraction of triterpenoids and other fractions with low polarity [164]. Despite its high efficiency, this method raises serious environmental concerns due to the use of organic solvents, prompting the search for more sustainable alternatives, such as enzymatic hydrolysis [161]. A promising sustainable alternative is mild-alkaline extraction, which has been shown to provide higher yields than hot-water extraction while preserving the structural integrity of proanthocyanidins [166]. To maximize the use of raw materials, sequential extraction can be used. This method involves the successive use of different solvents or extraction techniques. An example is the extraction of betulinic acid from sycamore bark using organic solvents, followed by further processing of the residue to extract polysaccharides with anticancer properties [101]. This economical method enables the effective utilisation of waste materials, thereby increasing the overall efficiency of the process. After solvent extraction, post-extraction waste is generated, comprising residual biomass and by-products. The choice of extraction method is crucial and depends on many factors, including the desired compounds, their purity, yield, and environmental goals. While solvent extraction often yields products with higher purity, HWE offers a more sustainable approach, which is becoming increasingly important in the face of growing environmental requirements.

#### 4.1.3. Alkaline Extraction

The alkaline bark extraction method is a process that utilises alkaline solutions to extract valuable components, including tannins, proanthocyanidins, and betulin, from various types of bark. This method is widely used in the food, pharmaceutical, cosmetic, and feed industries, highlighting its economic importance. Extraction is carried out at an elevated temperature (80–100 °C) for 5–30 min, which allows for the recovery of over 60% of the target compounds [129,130]. Although this method is very effective, it can lead to structural changes in the extracted materials, which may affect their functionality [167]. After extraction, acid is added to the solution to precipitate valuable components, which facilitates their separation [165]. The extracted compounds have a wide range of applications. For example, tannins, obtained from the bark of coniferous trees, are used in leather production, medicine, and as adhesives [82]. Alternatively, betulin, a triterpenoid with pharmacological properties, is extracted from birch bark and utilised in medicine and veterinary medicine [102]. The alkaline extraction method generates waste that can be recycled and reused. The remaining bark pulp can be further processed, for example, by bleaching, or it can be returned to the pulping process [165]. The residue from tannin can be used to manufacture cellulose products or biodegradable products for the production of biological products [82]. Additionally, during the extraction process, betulin, suberin, fatty acids, and other compounds are isolated, which can also be utilised [184]. Proper waste management, when integrated with other industrial processes such as chemical dissolution, exemplifies a sustainable approach to the economy.

### 4.2. Modern Methods

#### 4.2.1. Organosolv Extraction

Organosolv is a type of pulping and extraction method that uses organic solvents (e.g., methanol, ethanol, acetone, or mixtures of those with water), uses elevated temperature and pressure, and acts as a solvent to solubilize lignin and hemicelluloses. It produces high-purity lignin at low levels of contaminants, which are advantageous for many advanced material and chemical applications [100,169,185,186]. The organosolvent extraction method is a promising technique for isolating lignin and other valuable compounds from lignocellulosic biomass. It uses organic solvents, often in combination with water and acids, to dissolve lignin while preserving its structural integrity, making it suitable for further applications. The process typically involves treating lignocellulosic materials with a mixture of solvents, such as dioxane/water or water/1-butanol, under mild conditions [168]. The process can be further enhanced by using Lewis acids or organic esters, which improve lignin solubility and extraction efficiency [135,136]. An important advantage of organosolvent extraction is the ability to fractionate the extracted lignin into homogeneous fractions, which facilitates investigation of its structural characteristics and enhances its usefulness [168]. Techniques such as gel permeation chromatography (GPC) are commonly used to analyze and quantify the purity and molecular weight distribution of extracted lignin [168]. The extracted lignin can then be used as a renewable source for the production of aromatic compounds, biofuels, and dyes for polymers [169]. Additionally, this method facilitates the recycling of solvents, thereby contributing to a more sustainable extraction process [169].

Liu et al. [84] and Kasangana et al. [186] conducted analyses of bark processing (pine, oak, maple) in the organosolv process, often using ethanol or preliminary water extraction. Their work demonstrated effective bark fractionation, allowing for the isolation of crude lignin—usually rich in polyphenols, suberin compounds, and carbohydrates—and the recovery of sediments with increased carbohydrate content. These researchers also highlighted the potential of bark to be transformed into precious intermediates such as furfural and 5-hydroxymethylfurfural (HMF).

Following this approach, Grzybek et al. [85] focused on the impact of preliminary hot water treatment on the yield and properties of lignin from the bark of European trees (spruce, larch, and beech), which were also obtained using the organosolv method. Their research proved that pretreatment has a significant impact on the chemical composition and antioxidant properties of the obtained extracts.

Pals, M. et al. described the mild organosol delignification of poplar and willow bark residues after prior removal of extracts. The results of their work indicate the possibility of effectively obtaining fractions rich in lignin (up to 41%) and carbohydrates (up to 80%). These fractions represent promising raw materials for the production of biopolymers, such as polyurethanes or epoxy resins, as well as for the production of bioethanol, which is in line with the idea of a circular economy and sustainable development [50,138]

#### 4.2.2. Supercritical Carbon Dioxide (ScCO_2_) Extraction

Bark extraction using supercritical carbon dioxide (ScCO_2_) is an effective and environmentally friendly method that enables the selective extraction of precious bioactive compounds [170]. This process is typically carried out at temperatures ranging from 40 to 100 °C and at pressures of up to 62 MPa, allowing for the optimisation of yield and recovery of the desired substances. To increase extraction efficiency, especially for specific compounds, co-solvents such as ethanol can be utilised [171]. This method is particularly valued for eliminating toxic organic solvents, which reduces environmental pollution, and for the possibility of targeted extraction of compounds, such as polyphenols or essential oils, with beneficial health properties [141,142,172]. Examples of applications include the extraction of antibacterial compounds from the bark of *Stereospermum fimbriatum* [173] and polyphenols from Norway spruce (*Picea abies*) [171].

Extraction using supercritical carbon dioxide (ScCO_2_) offers additional benefits, including modification of the surface composition of biomass, which can enhance the efficiency of subsequent processes, such as pyrolysis [187]. Despite its many advantages, this method has limitations, as it may not be suitable for all compounds, particularly those that are sensitive to high pressure or temperature. Another key factor for the wider application of this technique is the balance between extraction efficiency and cost-effectiveness on an industrial scale [74].

After the ScCO_2_ extraction process, post-extraction waste is produced in the form of solid residual biomass and unextracted components. This waste is not useless, as it may contain precious substances that can be further processed. Residual biomass, rich in cellulose and lignin, can be used for bioenergy production or as a raw material in other chemical processes. Starch-rich residues can be converted into chemical compounds, such as hydroxymethylfurfural (HMF) [174]. Furthermore, unextracted fatty acids can be used to produce biodiesel, and other bioactive compounds can be used for pharmaceutical and cosmetic purposes [175]. 

#### 4.2.3. Enzymatic Hydrolysis

Enzymatic hydrolysis of tree bark is a promising method for converting lignocellulosic biomass into valuable products such as biofuels and biochemicals. This process involves breaking down complex carbohydrates into simpler sugars, which can then be fermented and utilised. To maximise sugar yield, effective pretreatment is key. For example, microwave-assisted pretreatment with dilute acid significantly reduced the lignin content and increased the crystallinity of cycad bark, which improved the efficiency of enzymatic hydrolysis. Optimal enzyme dosage is also crucial for cost-effectiveness. For sago bark, a cellulase concentration of 24 FPU/g (where FPU is the amount of enzyme required to liberate 1 µmol of glucose from cellulose) was considered optimal [114]. Different types of bark require different strategies. *Eucalyptus globulus* bark was successfully hydrolyzed using a commercial enzyme consortium, achieving 76% conversion at high solids loading [113]. In turn, the application of xylanolytic extracts from *Pseudozyma* sp. to *Eucalyptus dunnii* bark highlighted the importance of specific enzymes, such as xylanase and acetoxylanesterase, in xylan degradation [176]. Despite its great potential, the cost of enzymes, scalability, and the need for more effective pretreatment methods remain challenges, requiring further research and technological advancement.

#### 4.2.4. Energy-Assisted Methods—Steam Explosion and Microwave- and Ultrasound-Assisted Extraction

Energy-assisted extraction methods, such as steam explosion, microwave-assisted extraction (MAE), and ultrasonic-assisted extraction (UAE), represent an innovative approach to obtaining valuable compounds from tree bark. These methods increase the efficiency and effectiveness of extraction processes. Microwave-assisted extraction (MAE), which utilises microwave energy to heat polar molecules rapidly, significantly reducing the extraction time and solvent consumption, aligning with the principles of green chemistry [177]. Ultrasound-assisted extraction (UAE), on the other hand, uses ultrasonic waves for cavitation, which facilitates the release of bioactive compounds from the ruptured cell walls of plants [178]. Studies on willow bark have shown that MAE, especially in combination with ethanol, effectively extracts phenolic compounds, flavonoids, and tannins with strong antioxidant and antibacterial properties [179]. Additionally, MAE has been used to extract salicin and proanthocyanidins from aspen and pine bark, achieving higher concentrations than traditional methods [180]. The combination of both methods—simultaneous microwave and ultrasonic extraction—has been demonstrated to significantly increase extraction efficiency and enhance antioxidant properties while reducing the process time in studies on the bark of European larch (*Larix decidua*) [181]. Although traditional extraction methods are still widely used due to their simplicity, the integration of advanced energy-assisted technologies represents a promising direction for the future of sustainable and efficient extraction processes.

#### 4.2.5. Energy and Scalability Constraints in Green Extraction Methods

Advanced green extraction technologies present distinct environmental advantages; however, each one encounters significant challenges during scale-up. Supercritical CO_2_ offers excellent selectivity and produces solvent-free extracts, but its substantial energy requirements for CO_2_ compression and recycling continue to hinder industrial adoption [188,189,190]. Subcritical water extraction is effective, adjustable, and solely reliant on water, but it necessitates precise energy and temperature optimization to be viable at a larger scale [191]. Deep eutectic solvents are biodegradable and highly selective, though their high viscosity and recycling challenges significantly limit industrial feasibility [192,193]. Organosolv extraction yields high-purity lignin and cellulose fractions, but the process critically depends on efficient solvent recovery, which drives costs and determines environmental performance [194,195]. Overall, despite strong alignment with green-chemistry principles, improvements in energy efficiency, solvent recovery, and process integration are essential for broader industrial adoption of these methods [196].

### 4.3. Life Cycle and Techno-Economic Considerations

Recent studies emphasize the need for integrated environmental and economic assessment of bark extraction and valorization processes using life cycle assessment (LCA) and techno-economic analysis (TEA). Integrated LCA–TEA frameworks provide a holistic perspective on sustainability performance, identifying both environmental hotspots (e.g., solvent recovery, energy use) and economic bottlenecks (e.g., high CAPEX/OPEX, separation steps) in bark-based biorefineries [106,119,197,198,199,200]. Modern green extraction techniques such as microwave-assisted, supercritical CO_2_, and deep eutectic solvent (DES) methods have shown considerable promise in improving yield and reducing environmental impacts [201,202]. Despite progress, methodological inconsistencies—particularly in system boundary definitions, allocation methods, and impact categories—remain a major challenge. Recent LCA studies increasingly adopt ISO 14040/44 standards and full value-chain approaches while integrating uncertainty analysis to address these gaps [203,204,205]. On the TEA side, the use of machine learning models (e.g., Random Forest, Gradient Boosting) is becoming more common for cost prediction and process optimization, offering alternatives to traditional Monte Carlo methods [206,207,208,209]. From an environmental standpoint, strategies such as renewable energy integration and high ethanol recycling rates have been shown to reduce global warming potential (GWP) by up to 83% [197,210]. Economically, however, feedstock heterogeneity and limited industrial-scale data remain barriers to reliable techno-economic conclusions. To address this, researchers have called for more primary, feedstock-specific LCA/TEA studies and harmonized methodological frameworks [41,211,212].

## 5. Waste Extraction

### 5.1. Lignocellulosic Waste

**Figure 3:**
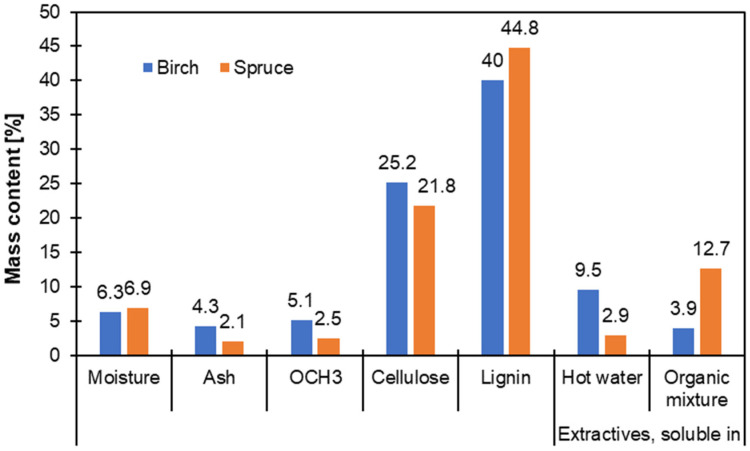
Share of Main Components in Birch and Spruce Tree Bark (own elaboration).

Bark extraction waste is the most common type of waste from wood biomass extraction and is a promising raw material in the circular economy. This waste, representing 75% to 95% of the original biomass, is rich in cellulose, hemicellulose, and lignin, which are the basic structural components of wood and bark. The percentage share of the main components in wood bark (polyphenols, lignin, cellulose) is illustrated in Figure 3.

These compounds are insoluble in water and often remain in the waste after the extraction process. The chemical composition of bark waste varies depending on the layer of bark. The outer bark has a higher content of cellulose (33.4%), lignin (31.7%), and hemicellulose (26.2%) compared to the inner bark [213]. These natural polymers can be reused to produce valuable materials and chemicals [214,215].

There are various methods for valorizing lignocellulosic waste. One of these methods is alkaline extraction, which effectively recovers lignin and subsequently enables cellulose digestion [213]. Another method is fast pyrolysis, which converts waste into biochar and bio-oils. For example, pyrolysis of cork oak bark produces biochar with high energy value (25.8–30.1 MJ kg^−1^) and nutrient content, making it useful as a soil improver [156,157]. Lignocellulosic waste can also be utilized in the production of chipboard, where it enhances the material's mechanical properties, provided that the amount is not excessive [36]. In addition, these wastes are a source of raw materials for the production of biofuels (such as bioethanol or biodiesel), biochemicals (e.g., bioplastics), and advanced materials such as activated carbon or polyurethanes [115,216,217,218,219].

The valorization of bark waste contributes to a circular economy by minimizing waste and reducing dependence on non-renewable raw materials [24,161]. These processes can also lead to energy savings and lower CO_2_ emissions, which increases overall sustainability in the industry [157,162].

### 5.2. Resin and Wax Waste

Resin and wax residues generated during tree bark extraction represent a chemically diverse fraction composed primarily of fatty acids, resin acids (abietic, dehydroabietic, isopimaric), triterpenes, sterols, polyphenols, suberin, and long-chain aliphatic alcohols [116,117,118,220]. These lipophilic compounds primarily occur in coniferous bark and are typically recovered through solvent extraction, supercritical CO_2_ extraction, or microwave-assisted extraction (MAE). Cascade or sequential extraction schemes can improve recovery efficiency and selectivity [221,222,223,224]. The resulting fractions exhibit strong hydrophobic, adhesive, and antimicrobial properties, enabling their use in bio-based adhesives, coatings, composites, and polymer formulations, as well as in the synthesis of renewable surfactants and lubricants [41,105,106,107]. Integrating resin and wax recovery into bark biorefinery systems supports the development of multi-product valorization pathways and contributes to the circular bioeconomy by maximizing feedstock utilization and reducing dependence on fossil-derived raw materials [41,105,107,119]. However, challenges remain due to chemical heterogeneity, fractionation complexity, and process scalability, which limit the reproducibility and industrial implementation of these technologies [106,225,226]. Future work should focus on the standardization of analytical and extraction protocols and on the optimization of green fractionation methods to enable efficient, large-scale recovery and upgrading of resin and wax residues as integral components of sustainable, zero-waste bark biorefineries.

### 5.3. Wastes with Residual Active Compounds

In addition to resin- and wax-rich residues, bark extraction processes generate solid and liquid fractions that still contain significant amounts of bioactive compounds. These include polyphenols (total phenols, flavonoids, tannins, proanthocyanidins), triterpenic acids (ursolic, betulinic, oleanolic), sterols (β-sitosterol), fatty acids, and suberin-related molecules that retain strong antioxidant, antimicrobial, and UV-protective properties [103,104,120,227]. The presence of these compounds demonstrates that bark residues, traditionally regarded as waste, represent a valuable secondary feedstock for the recovery of functional chemicals applicable in the pharmaceutical, nutraceutical, and cosmetic industries.

The recovery of residual actives has progressed beyond conventional maceration and Soxhlet extraction toward green and hybrid extraction techniques, including ultrasound- and microwave-assisted extraction, supercritical fluid extraction, enzyme-assisted hydrolysis, and the use of natural deep eutectic solvents (NADESs) are classified as a group of green solvents that consist of a mixture of a hydrogen bond donor (e.g., urea, organic acids) and a hydrogen bond acceptor (commonly choline chloride) to produce a eutectic that has a melting point considerably lower than the individual components. DESs are biodegradable, inexpensive, and highly tunable, making them increasingly popular for selective extraction of lignin, polyphenols, and other bark constituents [1,70,228,229,230,231]. These advanced processes enhance extraction yield, selectivity, and energy efficiency while maintaining the bioactivity of recovered compounds. Optimization of solvent polarity, temperature, extraction time, and solvent-to-solid ratio, often supported by statistical tools such as response surface methodology, enables the efficient recovery of thermolabile phenolics and triterpenoids [232].

Within cascade and zero-waste biorefinery frameworks, these bioactive-rich residues are increasingly integrated into multi-product valorization schemes. Sequential extraction strategies allow for the recovery of diverse compound classes, whereas the remaining lignocellulosic matrix can be converted into biofuels, biochars, or biofertilizers, thus supporting near-zero-waste utilization of bark biomass [41,197,233,234,235,236,237]. This integration not only improves the overall economic efficiency of bark processing but also enhances its environmental sustainability, aligning with the principles of the circular bioeconomy.

Despite recent advances, substantial knowledge gaps remain. Challenges include fractionation complexity, the scaling of green extraction and catalytic upgrading methods, and the limited analytical throughput for real-time quality control [106,211,238]. Moreover, comprehensive life cycle and techno-economic assessments of integrated extraction–valorization systems are still scarce, and few studies have explored the full potential of post-extraction residues containing active compounds. There is also a lack of standardized analytical protocols and integrated process models linking bark chemistry, extraction efficiency, and product performance. Addressing these research gaps through systematic experimental optimization, modeling approaches, and process integration will be crucial to establishing robust, scalable, and sustainable pathways for the recovery and utilization of residual bioactive compounds from bark extraction wastes [105].

### 5.4. Future Perspectives and Research Gaps

The efficiency of bark extraction is currently constrained by several technology-specific limitations associated with extraction mechanisms, solvent systems, and product purity. Advanced methods such as microwave-assisted ionic liquid extraction offer improved yields but require precise control of ionic liquid structure, temperature, and time to avoid inefficiencies [239]. Similarly, autoclave-based extraction, while effective for selected bark types, still involves multiple energy-intensive operations such as repeated extraction and centrifugation that hinder scale-up [240]. Solvent selection remains critical, as the anionic composition of ionic liquids strongly influences extraction selectivity and efficiency, demanding careful optimization for different bark chemistries [229]. High-pressure and high-temperature solvent systems can further enhance the recovery of valuable compounds, but such conditions also introduce degradation risks that must be balanced against yield [69,241]. Process challenges are compounded by the presence of non-process elements (NPEs) such as Ca, Mg, Si, and Cl, which contribute to corrosion and scaling during operation [242], and by seasonal variability in bark moisture that directly affects extraction outcomes [243]. From an industrial perspective, the costs associated with debarking, although beneficial for reducing NPEs, remain a major economic bottleneck, highlighting the need for cost-optimized pretreatment strategies [242]. Green extraction technologies, particularly those employing pure water under high pressure, show strong sustainability potential but still face barriers to industrial adoption due to technological complexity and energy demand [241]. Despite advances in extraction technologies, the intrinsic chemical heterogeneity of bark further complicates process optimization and directly influences achievable yields and product quality. In addition to these overarching technological and economic constraints, one of the most critical aspects shaping future research is the intrinsic chemical variability of bark, which continues to limit process standardization, scalability, and reproducibility across species and extraction methods. The pronounced chemical variability of tree bark resulting from species-specific composition, anatomical differences, and environmental conditions poses a persistent challenge for achieving consistent extraction yields and product quality [103,238,244,245]. This heterogeneity necessitates the development of adaptive and data-driven extraction strategies that can respond to variable phenolic profiles and optimize the recovery of bioactive compounds. Future research should therefore focus on tailoring solvent systems and process parameters to the chemical characteristics of individual bark types, supported by statistical optimization tools such as response surface methodology (RSM) to minimize resource consumption and maximize yield [103]. Green extraction technologies, including subcritical water and supercritical CO_2_ extraction, hold particular promise for future implementation because they eliminate the need for hazardous solvents and enable selective recovery of thermolabile bioactives [71,246]. Likewise, microwave- and ultrasound-assisted extraction techniques significantly enhance mass transfer rates, reducing processing time and energy consumption while maintaining high antioxidant activity of extracts [162,232]. Integrating these approaches within cascade valorization frameworks where lignin, cellulose, and extractives are sequentially recovered could improve economic viability and move bark processing closer to zero-waste industrial models [119,162]. In the broader perspective, coupling these optimized extraction systems with machine learning-based predictive models could enable real-time adjustment of parameters to account for bark variability. This integration represents a key research direction in achieving scalable, energy-efficient, and environmentally responsible bark valorization technologies.

Looking ahead, future investigations should not only focus on improving extraction efficiency but also on developing integrated cascade valorization models, in which both the primary extracts and the post-extraction residues are treated as valuable feedstocks. Current research rarely addresses the composition, structure, and potential reuse of solid and liquid residues generated during organosolv, alkaline, or suberinic acid extraction, despite these fractions containing high concentrations of lignin, polysaccharides, and lipophilic compounds with functional properties comparable to those of the main products. Establishing standardized methodologies for their characterization and valorization will be crucial for advancing bark biorefinery concepts.

Such an approach directly supports the broader objective of developing sustainable, zero-waste bark processing systems, in line with the cascade model proposed in this project. By integrating primary extraction and residue valorization, future bark biorefineries could achieve greater material efficiency, lower environmental impact, and provide a comprehensive knowledge base for the effective and circular utilization of *Betula*, *Picea*, and *Pinus* bark resources. 

## 6. Conclusions

Tree bark represents a valuable yet underutilized source of lignocellulosic and bioactive compounds. Considerable progress has been made in understanding its chemical composition and optimizing extraction techniques for lignin, tannins, suberin, and polyphenols. However, research has largely focused on primary extraction products, while post-extraction residues remain insufficiently explored despite their high content of functional molecules and structural polymers. These residues have strong potential for use in bio-based materials, energy generation, and green chemical synthesis. Major knowledge gaps persist in process standardization, the scalability of green extraction technologies, and comprehensive life cycle assessments. Future studies should emphasize integrated cascade valorization models that recover both extracts and residues, improving resource efficiency and environmental sustainability. Such an approach will advance the transition toward circular bioeconomy pathways and promote the full utilization of bark biomass in sustainable material development.

## Figures and Tables

**Table 1:** Structural and Chemical Diversity of Tree Bark Across Species: Implications for Extraction Potential.

Aspect	Angiosperms vs. Gymnosperms	Intra- Species Variation	Chemical Composition	Extraction Potential	Environmental/Phylogenetic Influence
Bark Thickness	Angiosperms: thinner, variable; Gymnosperms: thicker, especially the outer bark [46,53,54]	Varies with stem diameter, fire regime, climate [55,56]	−	Thicker bark often yields more extractives [57,58]	Fire, climate, soil, altitude [59]
Microstructure	Vessel presence in angiosperms; tracheids in gymnosperms [60,61]	Cell dimensions, microfibril angle, tissue allocation varies along the stem [62]	−	Microstructure affects mechanical extraction [63]	Genetic and environmental factors [64]
Chemical Profile	Species-specific phenolics, triterpenes, sterols [65]	Layer-specific (inner vs. outer bark) [66,67]	Lignin, suberin, extractives [68,69]	Extraction yield depends on compound type and location [70,71]	Age, geography, growth conditions [72,73]
Extraction Efficiency	−	−	Varies by method: supercritical CO_2_, subcritical water, ultrasound [74]	Cosolvent addition, temperature, solvent polarity critical [75,76]	−
Knowledge Gaps	Weak phylogenetic signals for thickness; incomplete structure-chemistry correlation [46,77]	High intra-/inter-specific variation [73,77]	Lignin/suberin structure poorly characterized [78,79]	Standardized protocols are lacking [68]	−

**Table 2:** Major chemical constituents of tree bark and their high-value applications.

Group of Compounds	Representative Components	Example Tree Species	Functional Properties	High-Value Applications
Polyphenols	Tannins, flavonoids, proanthocyanidins [43,81,82]	*Picea abies*, *Pinus radiata*, *Betula* spp., *Abies alba* [43,44,45,47]	Antioxidant, antimicrobial, protein-binding, UV-protective properties [47,80]	Natural adhesives, preservatives, nutraceuticals, anti-aging cosmetics, antioxidant extracts [80,81,99]
Lignin	Guaiacyl, syringyl and H-type lignin [84,85,86]	*Picea abies*, *Larix* spp., *Fagus sylvatica*, *Betula* spp. [84,85,86]	Aromaticity, thermal stability, antioxidant properties, polymer network formation [68,86]	Bio-based polymers (epoxy resins, polyurethanes), carbon-fiber precursors, UV-absorbers, composite fillers [84,89,100]
Suberin and suberinic fatty acids	ω-hydroxyacids, α,ω-dicarboxylic acids, long-chain fatty alcohols [90,93,96,98]	*Betula pendula*, cork oak (*Quercus suber*), *Pinus* spp. [90,93,94]	Hydrophobicity, chemical resistance, barrier properties, antimicrobial activity [92,96]	Biopolyesters, biodegradable coatings, barrier materials, biosorbents, green composites [90,92,95]
Triterpenes	Betulin, betulinic acid, oleanolic acid, ursolic acid [91,101,102]	*Betula* spp., *Pinus* spp., *Salix* spp. [44,45,91]	Antiviral, anti-inflammatory, cytotoxic, antioxidant activity [102,103]	Pharmaceuticals (anticancer, antiviral), dermatological cosmetics, bioactive extracts [102,104]
Sterols	β-sitosterol, stigmasterol [91,105]	*Pinus nigra*, *Pinus brutia*, *Picea* spp. [91,101]	Hypocholesterolemic activity, oxidative stability [105,106]	Functional food additives, nutraceuticals, cosmetic stabilizers [105,106,107]
Polysaccharides	Cellulose, hemicellulose, pectins [108,109,110]	*Pinus* spp., *Salix* spp., *Betula* spp., *Eucalyptus* spp. [111,112,113]	Gel-forming capacity, biodegradability, structural reinforcement [108,114]	Hydrogels, bio-polymers, bio-packaging, feedstock for bioethanol, composite additives [108,113,115]
Resin and wax components	Resin acids (abietic, dehydroabietic), long-chain alcohols, fatty acids [116,117,118]	Conifer bark (*Pinus* spp., *Picea* spp.) [116,118]	Hydrophobicity, adhesive properties, antimicrobial activity [105,106]	Natural coatings, bio-adhesives, surfactants, lubricants, polymer additives [105,107,119]
Lipophilic extractives	Phytosterols, long-chain fatty acids and esters [103,116,118]	*Pinus* spp., *Picea* spp., *Betula* spp. [103,118]	Antioxidant, antimicrobial, hydrophobic properties [103,120]	Cosmetics, bio-lubricants, nutraceuticals, green surfactants [104,105,120]

**Table 3:** Bark Utilization Strategies and Their Benefits Across Different Sectors.

Sector	Bark Utilization Strategy	Key Benefits/Properties
Materials Industry	Production of wood-based boards (e.g., particleboard)	Using natural binders (tannins, polyphenols) to replace synthetic adhesives, resource optimization, sustainable development. [124,125,126,127,128]
	Production of insulation boards	Better thermal conductivity and heat storage capacity, excellent sound-absorption properties, the possibility of creating panels without using adhesives. [5,97,98,129]
	Processing into porous materials	Creating structures with a variety of properties through mechanical foaming. [130]
Farming and Gardening	Use as substrates and soil improvers.	Supports plant growth, improved aeration and drainage, significant increase in wheat yields and quality, potential to increase soil organic carbon content and cation exchange capacity. [23,100,101,104,131,132,133]
Energy Sector	Use as biomass for energy production	Contributes to reducing greenhouse gas emissions, high calorific value, and energy efficiency (direct burning). [48,105,108]
	Chemical Looping Combustion (CLC)	An effective and economical method of utilizing bark from the pulp and paper industry. [134]
	Gasification (in two-fluid steam gasifiers)	Allows for the production of synthesis gas (syngas) and subsequent thermal and electrical energy; the efficiency of converting biomass into biomethane can reach up to 65%. [106,107,135,136,137]
	Production of high-calorie energy briquettes	Provides an alternative fuel source (when combined with plastics and oil), helps with waste management. [138]
Dietary Supplements, Cosmetics and Pharmaceuticals	Production of cosmetics	Rich in bioactive compounds (polyphenols, flavonoids), strong antioxidant properties, effectively block UV radiation (sunscreens). [99,139,140]
	Production of dietary supplements	Recognized as potential sources of polyphenols that support health, aligns with the idea of a circular economy. [141]
Bark-derived lignin utilization strategy	Production of lignin-based polymers (e.g., rigid foams)	Bark-derived lignin enables the development of high-performance bio-polymers, renewable adhesive systems and advanced lignin-based materials, benefiting from its elevated phenolic content, reactive hydroxyl groups and suitability for producing bio-polyols [142,143,144,145,146]
	Manufacturing of bio-based adhesives	
	Development of functional materials (e.g., NIPUs)	

**Table 4:** Typical chemical composition of residues obtained from different bark extraction methods.

Extraction Method	Main Residue Components	Typical Characteristics/Composition Ranges	Key Remaining Bioactives
Hot Water Extraction (HWE)	Lignocellulosic matrix: cellulose, hemicellulose, lignin [108,109,110]	High ash content (up to 50%); partial hemicellulose solubilization [108,110]; increased structural polysaccharides [113]	Polyphenols and tannins remaining in the insoluble fraction [108,113]
Solvent Extraction	Cellulose, lignin, hemicellulose, suberin [43,81,82,91]	Reduced extractives fraction; retention of structural polymers; partial removal of lipophilic compounds [43,81]	Residual tannins, flavonoids and phenolics [43,82]
Deep eutectic solvents (DESs)	Cellulose-rich residues, lignin–DES complexes [92,95,96]	Partial delignification; strong DES–lignin interactions; high carbohydrate retention [95,96]	Phenolics and flavonoids trapped in the DES–biomass matrix [92,95]
Organosolv Extraction	High-purity lignin, cellulose pulp [84,85,86]	Sulfur-free lignin; improved cellulose accessibility; reduced hemicellulose fraction [85,113]	Polyphenols and low-molecular aromatics retained in residues [84,113]
Supercritical CO_2_ Extraction (ScCO_2_)	Lignin, cellulose, waxes, long-chain fatty acids [80,105,116]	Limited removal of polar extractives; residues contain waxes and lipophilic compounds [80,116]	Essential oils, triterpenes, phenolics not fully extracted [80,105]
Enzymatic extraction/ enzymatic pretreatment	Cellulose, residual lignin, mineral residues [111,112,114]	Enhanced accessibility of polysaccharides; selective removal of target components [112,114]	Polyphenols and oligosaccharides remaining in the solid phase [111,112]

**Table 5:** Comparison of Tree Bark Extraction Methods in Terms of Extraction Medium, Environmental Impact, and Main Products/Applications.

Extraction Method	Extraction Medium/Agent	Environmental Impact	Main Products/Applications
Hot Water Extraction (HWE)	Water (100–160 °C, optimal 140 °C) [119,120]	Very low [111]	Non-cellulosic polysaccharides (hemicelluloses, pectins), lignin [108,109,110]
Solvent Extraction	Organic solvents (acetone, methanol, ethanol, petroleum ether) [101]	High (toxic organic solvents) [161]	Tannins, lignin, phenolic compounds [101,163,164]
Alkaline Extraction	Alkaline solutions (NaOH, KOH)	Moderate [82,165,166,167]	Tannins, proanthocyanidins, botulin [129,130,132]
Organosolv Extraction	Organic solvent mixtures (ethanol/water, dioxane/water) with acids [168]	Low (solvent recycling possible) [169]	Lignin, aromatic compounds, biofuels, dyes [89,100,169]
Supercritical CO_2_ Extraction (ScCO_2_)	Supercritical CO_2_ (40–100 °C, up to 62 MPa) + co-solvents (ethanol) [139,140,170,171]	Very low [170,172]	Polyphenols, essential oils, antibacterial compounds [140,141,142,145,146,173,174,175]
Enzymatic Hydrolysis	Enzymes (cellulase, xylanase, acetoxylanesterase) [147,149]	Very low	Fermentable sugars, biofuels, biopolymers [113,114,176]
Energy-assisted Methods (Steam Explosion, Microwave-, Ultrasound-assisted Extraction)	Energy input (steam, microwaves, ultrasound) [150,153]	Low [177]	Phenolic compounds, flavonoids, tannins [151,152,154,178,179,180,181,182,183]

## Data Availability

Not applicable.
